# Diet with High Forage:Concentrate Ratio Improves Milk Nutritional Properties and Economic Sustainability of Buffalo Breeding

**DOI:** 10.3390/ani15142050

**Published:** 2025-07-11

**Authors:** Lorenzo Infascelli, Nadia Musco, Piera Iommelli, Giuseppe Vassalotti, Francesco Capezzuto, Fabio Zicarelli, Valeria Maria Morittu, Anna Antonella Spina, Federico Infascelli, Raffaella Tudisco

**Affiliations:** 1 Department of Economics and Law, University of Cassino and Southern Lazio, 03043 Cassino, Italy; lorenzo.infascelli@unicas.it; 2 Department of Veterinary Medicine and Animal Production, University of Napoli Federico II, 80100 Naples, Italy; nadia.musco@unina.it (N.M.); capezzutofrancesco190@gmail.com (F.C.); fabiozicarelli@gmail.com (F.Z.); infascel@unina.it (F.I.); tudisco@unina.it (R.T.); 3 Department of Medical and Surgical Sciences, Magna Græcia University of Catanzaro, 88100 Catanzaro, Italy; giuseppe.vassalotti@gmail.com (G.V.); morittu@unicz.it (V.M.M.); aa.spina@unicz.it (A.A.S.)

**Keywords:** high forage diet, buffalo milk, sustainability

## Abstract

The present study aimed to evaluate the impact of two dietary regimens, characterized by different forage-to-concentrate ratios, on feed intake, milk yield and composition, as well as economic and environmental sustainability in lactating buffalo cows. Group H was fed a high-forage diet, while Group L was fed a diet with a lower forage-to-concentrate ratio. Despite a lower energy value (Unit for Feed Lactation: UFL 0.89 vs. 0.91), the diet of group H met the animals’ nutritional requirements, with similar dry matter intake (DMI) and body condition scores observed in both groups. While there was no significant difference in milk yield, protein, and lactose, group H exhibited significantly higher milk fat content and an enhanced fatty acid profile, characterized by increased oleic acid, conjugated linoleic acids (CLAs), and omega-3 polyunsaturated fatty acids. These fatty acids have been linked to potential health benefits in humans, including anti-inflammatory, cardioprotective, and anticarcinogenic effects. From an economic perspective, the H group’s diet was found to be more cost-effective, resulting in EUR 0.46 savings per head per day and a higher yield of mozzarella cheese. From an environmental perspective, the high-forage diet reduced reliance on imported concentrates and irrigation-dependent crops, aligning with sustainable development goals. In conclusion, increasing the forage content in buffalo diets has been demonstrated to support animal performance, improve milk quality, reduce environmental impact, and enhance economic returns for producers.

## Simple Summary

The aim of this study was to compare two diets differing in forage:concentrate ratio administered to lactating buffalo cows divided into two groups—one high in forage content (Group H) and one lower in forage (Group L)—in order to evaluate the outcomes in terms of milk production and nutritional characteristics. Despite a different energy concentration in the diets, the groups had similar feed intake, body condition scores, and milk yield, meaning that nutritional requirements were met by the diets. Results from milk analysis showed no differences in protein and lactose content, whereas milk from group H had significantly higher fat content as well as healthier fatty acids, specifically conjugated linoleic acids and omega-3 polyunsaturated fatty acids. The diets were evaluated also from an economic perspective. The high-forage diet was more cost-effective and environmentally friendly, reducing the need for imported feed and water-intensive crops. Overall, the high-forage diet improved milk quality, supported sustainability, and increased economic benefits.

## 1. Introduction

In Italy, the production of buffalo milk is predominantly concentrated in specific regions, where it is destined primarily for the production of a fresh PDO Italian cheese [1]. Over time, there has been a steady increase in the number of buffaloes farmed in Italy, reflecting growing economic interest in the production of mozzarella cheese [2] as well as buffalo meat, whose high nutritional quality has been demonstrated [3]. Beyond Italy, buffalo farming is also extensively practiced in other regions, particularly in India and other Asian countries, where the animals are primarily used to produce milk for direct consumption [4].

Buffalo milk is the second-largest source of milk worldwide, with an annual production that exceeds 75 million metric tons [4]. Cow milk typically contains an average of 3–4% fat and 3.5% protein, whereas buffalo milk exhibits higher nutritional value, with an average protein content ranging from 4 to 4.5% and fat content of approximately 6–8% [5]. Consequently, when evaluated in terms of energy-corrected milk (ECM), the nutritional contribution of buffalo milk is almost double that of cow milk [4].

A number of significant physiological and digestive differences have been identified between buffaloes and cattle, particularly with regard to the nutrient utilization efficiency. Buffaloes have been shown to possess a more efficient urea cycle, which results in reduced nitrogen excretion [6]. The efficiency observed in buffaloes is attributable to several factors, including higher rumen cellulolytic activity and better fiber digestion [7]; higher rumen pH and a superior buffering capacity of saliva [8]; elevated rumen ammonia concentration, reflecting enhanced urea recycling via saliva [6], and greater nitrogen balance efficiency, due to the optimized utilization of ammonia nitrogen by ruminal bacteria. These factors collectively contribute to buffaloes’ ability to thrive on lower-quality diets while maintaining high milk productivity.

When formulating buffalo diets, the forage:concentrate ratio (F:C) constitutes a pivotal step, given its effect on both the animal’s physiology and milk production parameters. A higher F:C has been shown to enhance fiber intake, which in turn has been demonstrated to promote improved rumen function and milk quality [9,10]. Infascelli et al. [11] reported that this ratio exerts a significant influence on livestock sustainability, which represents a crucial element in addressing global challenges related to climate change, food security, and ecosystem protection. Indeed, efficient management of F:C in animal nutrition can significantly influence animal productivity and their environmental impact, contributing to the achievement of the 17 Sustainable Development Goals (SDGs) of the United Nations’ 2030 Agenda [12]. By integrating various environmental, economic, and social aspects, this approach promotes the conservation of natural resources, responsible and efficient food production, and the well-being of rural communities, encouraging the adoption of sustainable agricultural practices and reducing greenhouse gas emissions. Furthermore, a balanced F:C can optimize the production of high-quality food, contributing to food security and responsible and conscious nutrition [13].

Acosta Balcazar et al. [14] highlighted that diets richer in forage can improve the lipid profile of milk produced by cows, increasing its nutritional value. In fact, a high F:C leads to an increase in conjugated linoleic acid (CLA), which can reduce cardiovascular risks and generate general health benefits for consumers, contributing to SDG 3 (Good Health and Well-being). Additionally, the use of high-quality local forage protects farms from risks associated with possible fluctuations in costs or the limited availability of imported concentrates, ensuring safe and sustainable milk production even in cases of scarce resources (SDG 2—Zero Hunger). Consequently, by promoting the use of local forage and resources and reducing the need for imported concentrates, it is possible to improve the efficiency and sustainability of the entire production chain, increasing its self-sufficiency (SDG 12—Responsible Consumption and Production), significantly reducing the overall carbon footprint of farms (SDG 13—Climate Action), and achieving a greater safeguarding of natural ecosystems (SDG 15—Life on Land). Finally, greater productive and nutritional efficiency also results in increased profitability for farmers, contributing to SDG 8 (Decent Work and Economic Growth).

This article, therefore, aims to investigate how appropriate management of the forage:concentrate ratio can support the achievement of the SDGs, highlighting the existing connections between sustainable agricultural practices and global development goals. The present study aimed to evaluate the effect of different forage:concentrate ratios in the diet on buffalo milk production, focusing on both quantitative and qualitative aspects. It is hypothesized that increasing the forage component in the diet will enhance milk quality due to increased fiber intake, while also offering economic advantages. The study seeks to optimize this ratio, thereby balancing production efficiency with sustainability, and contributing to improved dairy management practices for buffalo farming.

## 2. Materials and Methods

The trial was carried out from March to June 2023 at a commercial buffalo farm located in the province of Caserta (Campania Region, Italy, 123 m a.s.l. 41°18′ N 14°15′ E, with 638 mm average rainfall and 15.3–25.5 °C mean temperature) and was approved by the Ethical Animal Care and Use Committee of the University of Napoli Federico II (Prot. 2019/0013729 of 8 February 2019).

### 2.1. Animals and Diets

Forty Italian Mediterranean buffalo cows were equally divided into two groups (H, high forage vs. L, low forage) homogeneous for parity (3.65 ± 0.5 vs. 3.81 ± 0.5), body weight (BW 619 ± 21 kg vs. 627 ± 26 kg), days in milk (DIM 29.5 ± 6 vs. 26.9 ± 5), previous milk yield (2140 ± 122 kg vs. 2200 ± 162 kg), and milk yield at the beginning of the trial (11.9 ± 2.1 kg vs. 12.1 ± 2.3 kg).

The groups were allocated to individual open yards with permanent bedding area (8 m^2^/head), exercise area (12 m^2^/head), feeding area (3.5 m^2^/head), and free access to water. The animals were individually fed total mixed rations (TMRs), differing between groups by their forage:concentrate ratio on a dry matter basis (H, 58:42 vs. L, 45:55, estimating corn silage as 50% forage and 50% concentrate), whose ingredients are reported in Table 1.

TMR refusals were collected daily to calculate the individual dry matter intake (DMI). The animals’ body condition score (BCS) was evaluated weekly according to a scale of 1 to 5, where 1 = emaciated, 2 = thin, 3 = average, 4 = fat, and 5 = obese [15].

### 2.2. Diet Analysis

Before feeding, samples of TMR were weekly collected from the feed fence, oven-dried at 65 °C, milled through a 1 mm screen, and analyzed for dry matter (DM), crude protein (CP), and ether extract (EE) contents (ID number: 2001.12, 978.04, 920.39 and 978.10, 930.05, respectively), as suggested by AOAC [16]. The structural carbohydrates were determined according to Van Soest et al. [17], while starch was assessed by the polarimetric method (Polax L, Atago, Tokyo, Japan), as per the official procedure (ISO 6493:2000 28) [18]. TMR nutritive value (UFL = 1700 kcal of net energy for lactation) was calculated as reported by INRA [19]. For the fatty acid (FA) profile of the TMRs, approximately 200 mg of fat was extracted as reported by Folch et al. [20], successively methylated according to Christie [21], and injected into a gas chromatograph (GC) with flame ionization detector (GC-FID TRACE 1310 system equipped with an AI 1310 Auto-injector/AS 1310 Autosampler; Thermo Fisher Scientific, Milan, Italy). The Omegawax 250 capillary polar column (Supelco, Bellefonte, PA, USA), 30 m × 0.25 mm, 0.25 μm (L × I.D., film thickness) was utilized with a temperature program of 100 °C for 5 min, 100–240 °C at 4 °C/min, and final isotherm at 240 °C for 20 min. Both the injector and detector temperature were 250 °C; injection volume, 0.5 µL; split ratio, 1:50. Carrier gas (He) at a flow rate of 1 mL/min; make-up gas (N2) flow, 40 mL/min; H2 flow, 35 mL/min; air flow, 350 mL/min. Data were processed using the Chromeleon™ Data System (Thermo Fisher Scientific, Milan, Italy) software (Version 7.2.9), and individual FAMEs identified by a comparison of the peak retention times with standards from Supelco (Merck KGaA, Darmstadt, Germany). The single FA concentrations were expressed as g/100 g, considering 100 g the total of all areas of the identified FAMEs.

### 2.3. Milk

Individual milk yield (MY) was daily registered (by using the TDM software, version 4.1), while individual milk samples (weighing the two daily milkings) were collected monthly and analyzed for chemical composition (fat, protein, and lactose) by using Milkoscan 133B calibrated for buffalo milk (Foss Matic, Hillerod, Denmark). For the study of the milk fatty acids profile, total fat was extracted with a hexanopropanol and isopropanol (3/2 *v*/*v*) mixture [22] and trans methylated according to the Christie [23] procedure as modified by Chouinard et al. [24]. The methyl esters were measured by a gas chromatograph (ThermoQuest 8000TOP. Thermo Electron Corporation, Rodano, Milan, Italy) equipped with a flame ionization detector and capillary column (CP-SIL 88 fused silica capillary column, 100 m × 0.25 mm internal diameter with 0.2 μm film thickness; Varian Inc., Walnut Creek, CA, USA), choosing the temperature ramp of 70 °C for 4 min → 13 °C/min → 175 °C for 27 min → 3 °C/min → 215 °C for 38 min → 10 °C/min → 70 °C. The injector and detector temperatures were 250 and 260 °C, respectively. Gas flows were the following: carrier gas (helium) 1 mL/min; hydrogen 30 mL/min; air 350 mL/min; make-up gas (helium) 45 mL/min. The fatty acid peaks were revealed by a comparison with a standard mixture of fatty acid methyl esters (Larodan Fine Chemicals, AB, Limhamnsgårdens, Malmö, Sweden). Finally, the identification of the isomers of conjugated linolic acid (CLA) was determined by comparing sample chromatograms with those of single purified isomers (CLA cis-9, trans-11; CLA trans-10, cis-12; CLA cis-9, trans-11; CLA trans-9, trans-11) (Larodan Fine Chemicals, AB, Limhamnsgårdens, Malmö, Sweden).

Finally, the mozzarella cheese yield (MCY) was calculated by the formula of Altiero et al. [25]:

MCY (kg) = [3.5 (% protein) + 1.23 (% fat) − 0.88]/100

### 2.4. Statistical Analysis

Diet data were analyzed by two-way ANOVA according to the model:

yijk = m + Di + Sj + DxSij + eijk

where yijk = single observation; m = general mean; Di = diet effect (i = H and L); Sj = sampling effect (j = I, II, …… IV); DxS = interaction between diet effect and sampling effect; eijk = experimental error.

Milk data were analyzed by two-way ANOVA according to the model:

yijk = m + Gi + Sj + GxSij + d(Gi) + eijk

where yijk = single observation; m = general mean; Gi = group effect (i = H and L); Sj = sampling effect (j = I, II, …… IV); GxS = interaction between group effect and sampling effect; d(Gi) = random effect of buffalo within group; eijk = experimental error.

The comparison among the mean values was carried out using Tukey’s test, and the differences were considered significant at *p* < 0.05.

All the analyses were performed using the procedure of JMP^®^ (version 14; SAS Institute, Cary, NC, USA).

## 3. Results and Discussion

### 3.1. Animals and Diet

BCS showed no significant differences between the groups. The diet fed to group H, formulated to have a higher forage:concentrate ratio, showed a significantly higher percentage of structural carbohydrate but lower protein, extract, and starch content than that fed to group L (Table 2), and its energy value was lower (0.89 vs. 0.91 UFL). Nevertheless, according to Terramoccia et al. [26], both TMRs’ protein and energy concentrations were suitable for lactating buffalo cows. The fatty acid profile was similar between the diets except for the content of ω6, which was significantly lower in diet H, and ω3, which was significantly higher in diet H compared to diet L.

No TMR refusals were detected. The DMI was similar between the groups (16.1 ± 2.1 kg vs. 16.6 ± 20 kg, for groups H and L respectively), despite the different forage:concentrate ratio. Indeed, the control of DMI is not regulated only by physical factors (i.e., high volume occupied by the forage fiber fraction with slower fermentation rate compared to the cell content) but also by metabolic and hormonal items [27]. In addition, the buffalo shows extended mastication mainly in the case of a fibrous diet and also due to the developed incisive teeth [28,29], as well as to the high cellulose fermentation rate by the rumen microorganisms [30,31,32]. Accordingly, in buffalo, no significant differences have been reported for in vivo organic matter digestibility, comparing diets with 80:20 vs. 50:50 forage:concentrate ratios [33]. Interestingly, Bai et al. [10], in an in vitro trial using yak rumen fluid as inoculum, found significantly higher degradation of the TMR dry matter and protein as the forage:concentrate ratio increased. Nevertheless, very different DMI has been observed for buffalo cows, varying from 2.2–2.6% [34] to 2.7–3.4% body weight [35].

### 3.2. Milk Productive Traits

Average milk yield, protein, and lactose were not different between groups (Table 3); in contrast, milk fat was significantly higher in group H (90.1 vs. 85.6 g/kg; *p* < 0.01). In addition, except for the lactose, these parameters were significantly affected by the month of sampling. The interaction between group and month of sampling was in each case not significant. All biological activities require energy; therefore, increasing the concentrate in the diet, due to its higher energy level compared to the forage, should result in increased milk yield [36]. Accordingly, Habib et al. [37] reported an increase in milk yield in lactating buffaloes with an additional amount of concentrate in the diet. In contrast, Purcell [38] found no influence on the productive performance of high-yielding cows fed basal diet ad libitum, either in early or mid lactation. In the present trial, the milk yield was similar between groups probably because, as reported above, both diets guaranteed the nutritive requirements of lactating buffalo cows. No significant differences in milk yield were observed by Yoder et al. [39] also, in dairy cows fed diets with a forage:concentrate ratio of 55:45 vs. 70:30. The significantly higher fat percentage observed in milk of the group fed the diet with a higher forage:concentrate ratio could be due to the higher NDF content of diet H, which in the rumen promotes the synthesis of acetate, a precursor for milk fat synthesis [40]. Similar results have been reported also in dairy cows either during the peak lactation period [41] or in mid lactation [42,43].

The effect of the dietary treatment on milk fatty acid profile, probably the most important factor to estimate the health properties of food, is reported in Table 4. Concerning the saturated fatty acids (SFA), milk of group H showed significantly (*p* < 0.05) lower capric (10:0 C) but higher undecanoic (11:0 C) acid than group L. A decrease in milk capric acid has been reported also by Le Doux et al. [44] in goats fed a diet with a high forage concentrate ratio. According to Shekhar et al. [45], capric acid could be useful in the therapy for some human brain disorders (epilepsy, affective disorders, or Alzheimer’s disease) due its activity on AMPA receptors, PPAR-γ, inflammatory/oxidative stress pathways, and gut dysbiosis, even if further research is needed to confirm this efficacy. Significantly higher levels of undecanoic acid have been observed also in milk of dairy cows fed pasture vs. concentrate based diets by Clarke at el. [46]. Very interestingly, this fatty acid has been proved to inhibit the proliferation and clonogenic ability of cancer cells [47]. The oleic acid (C18:1 cis 9) was significantly higher in milk of group H (24.07 vs. 22.79 g/100 g fat, for group H and L, respectively; *p* < 0.05). A number of studies demonstrated the healthy properties, particularly the anti-atherogenic ones, of dietary oleic acid, very recently summarized by Lu et al. [48]: significant reduction of plasma total cholesterol, very low-density lipoprotein, and low-density lipoprotein by inhibiting the activity of their hepatic receptors, and a significant reduction of plasma triglyceride concentrations. Thus, the intake of oleic acid contributes to a significant reduction in both cardiovascular and coronary heart disease prevalence. Chung et al. [49] observed a higher concentration of oleic acid in milk from dairy cows fed organic (higher in forage) vs. conventional diets. According to Hanuš et al. [50], this result should be due to a negative energy balance (NEB) mainly in early lactation; indeed, oleic acid is the predominant fatty acid in adipocytes thus principally released through lipolysis. However, this phenomenon did not occur in the present trial because the BCS was similar between the groups. In addition, the decrease in short-chain FAs specific for NEB was not observed. Total CLA as well as the c9 t11 and t10 c12 isomers were higher in the milk of group H. The c9t11 represents a byproduct of the biohydrogenation of dietary linoleic acid to stearic acid (C18:0) by the rumen microbes [51]; additionally, milk CLA results from the desaturation of trans vaccenic acid (trans-11C18:1), an intermediate product of the rumen biohydrogenation of polyunsaturated fatty acids in the mammary gland by stearoyl-CoA desaturase [52]. According to Aguerre et al. [53], the higher forage:concentrate ratio affects rumen activities and metabolic state (i.e., higher rumen pH) and, consequently, improves the activity of the ruminal microbes. Increased milk CLA has been observed also in sheep [54], dairy cows [55], goats [11], and buffalo milk [56] fed diets with a high forage:concentrate ratio. The healthy characteristics of CLA include anticarcinogenic, antiobesity, antidiabetic, and antihypertensive activities [57,58]. According to Shultz et al. [59], CLAs are cytotoxic to human cancer cells, inhibiting the proliferation of human melanoma, colorectal, and breast cancer cells in vitro. Blankson et al. [60] observed weight loss, lower serum leptin concentrations, and less body fat in patients treated with dietary CLA. In the present trial, PUFAs, particularly those of the omega-3 series, were higher in milk of group H. This result agrees with a number of other studies carried out comparing diets with different forage:concentrate ratios in dairy cows [50], goats [61], sheep [62], and buffaloes [63]. According to Chilliard et al. [64], the concentration of different PUFAs in milk is mainly affected by animal diet, particularly by the forage:concentrate ratio, as forage is richer in linolenic acid (C:18:3 n3), and concentrate richer in linoleic acid (C18:2 n 6). The intake of both omega-6 and omega-3 PUFAs is essential for humans, with the optimum ω6:ω3 ratio within 2.1 to 4.1 [65], considering the balanced effects between pro-inflammatory, pro-aggregant, and immunosuppressive activities (ω6) and anti-inflammatory, antiaggregant, and non-immunosuppressive activities (ω3). Unbalanced diets could determine mitochondrial dysfunctions with consequent cellular damage caused by the excessive generation of reactive oxygen species relative to natural antioxidant activity [66].

### 3.3. Economic Analysis

The diet with the higher F:C ratio was economically advantageous; indeed, the cost of the ration (Table 5) in group H was lower than that for group L, with a mean savings of EUR 0.46/head/day (EUR 9.2/day/group). In addition, using the equation suggested by Altiero et al. [25], the mozzarella cheese yield was higher in group H (25.1% vs. 24.3% for group H and L, respectively) with 1.4 kg/day more mozzarella produced with milk from group H (Table 6). Therefore, in 120 days of the trial, group H yielded 168 kg more mozzarella cheese, with a ration cost savings of EUR 1104. Finally, the higher mozzarella cheese yield leads to lower costs for whey disposal.

The cost of corn silage, alfalfa hay, and polyphyte hay was determined at harvest compared to the local market; thus, it did not change during the whole trial. That of corn meal was determined monthly using the data collected by the Ager (Borsa Bologna https://www.agerborsamerci.it/listino-borsa/settimanale-ager/, (accessed on 17 May 2023), and the costs of concentrate and hydrogenated fats were collected monthly by a wholesale marketing company.

The diet with a higher F:C ratio was more sustainable from the environmental point of view: the higher amount of hay decreased that of concentrate, which, particularly when constituted by genetically modified ingredients, is imported from continents other than Europe; the lower use of corn silage reduced water and energy consumption for irrigation. Accordingly, Benchaar et al. [67], examining and evaluating the effectiveness of different nutritional strategies to reduce methane production in ruminants, found that the use of more easily digestible and less processed forage led to a reduction in methane production by −15% and −21%, contributing to SDG 13. Krizsan et al. [68] attempted to identify, from a scientific perspective, the challenges and opportunities to make Swedish dairy production more sustainable by improving resource efficiency, reducing environmental impact, and adapting to future climate mitigation and food security needs. The study found that optimizing forage use, particularly grass—the main agricultural crop in Sweden—is essential for enhancing the sector’s productivity.

## 4. Conclusions

The findings of this study demonstrate that a higher forage-to-concentrate (F:C) ratio in the diet of lactating buffalo cows can be an effective feeding strategy without compromising productive performance. Despite its lower energy density, the high-forage diet (group H) ensured similar dry matter intake and milk yield compared to the lower-forage group (group L), indicating that buffalo cows can maintain intake and production levels even with fibrous diets, likely due to their unique rumen physiology. Notably, the high-forage diet resulted in a significantly higher milk fat content and a more favorable milk fatty acid profile. The increased concentrations of milk oleic acid, CLA, and omega-3 in the high-forage group are associated with various health benefits in humans, including anti-inflammatory, anti-carcinogenic, and cardioprotective properties. From an economic standpoint, the adoption of a high-forage diet resulted in a reduction in feed costs and an enhancement of cheese yield, thereby offering financial benefits to producers. From an environmental perspective, reduced use of imported and irrigation-demanding concentrates supports resource conservation and lowers greenhouse gas emissions. In conclusion, increasing the F:C ratio in buffalo diets can be regarded as a sustainable and profitable choice that benefits animal health, product quality, and the environment.

## Figures and Tables

**Table 1:** Ingredients (in kg as fed) in total mixed rations for groups H and L.

	H	L
Corn Silage	12.0	20.0
Alfalfa Hay	4.5	2.0
Polyphyte Hay *	4.0	2.0
Corn Meal	2.0	2.2
Commercial Concentrate **	3.5	4.7
Hydrogenated Fats	0.1	0.1

* *Phleum Pratense* L., *Lolium italicum* L., *Trifolium pratense* L. ** Soybean de-hulled grains, cotton meal, sunflower de-hulled grains, wheat bran, dried brewers grains, barley meal, molasses, CaCO_3_, NaCl, MgO, sodium bicarbonate, Vit A 60,000 IU, Vit D3 8000 IU, Vit E 90.00 mg, Iodium 4.00 mg, MnO 200.00 mg, Se 0.60 mg, Zn 200.00 mg.

**Table 2:** Body condition score (BCS), TMR dry matter intake (DMI, kg), chemical composition (g/kg DM), nutritive value (UFL/kg DM) and fatty acid profile, expressed as g/100 g of fat extract.

	March		April		May		June		*p* Value			RMSE
	H	L	H	L	H	L	H	L	Diet	Time	T × D	
BCS	3.2	3.1	3.3	3.2	3.3	3.2	3.3	3.3	0.456	0.154	0.364	0.07
DMI	16	16.4	16	16.5	16.1	16.7	16.1	16.6	0.485	0.485	0.254	0.21
CP	146.9	153.3	145.7	152.1	150.1	156.3	149.8	155.2	0.062	0.065	0.942	4.27
PDIE	99.2	103.3	99.1	103.1	98.6	102.9	98.9	103.2	0.564	0.127	0.745	3.21
PDIN	96.2	100.2	96.5	101.2	97.01	100.2	96.3	99.8	0.567	0.432	0.321	2.54
EE	35.9	45.7	36.3	46.5	36	46.2	36.1	46.7	0.456	0.652	0.645	1.32
NDF	395.1	379.3	399	379.3	397.8	385.3	396	381.7	0.048	0.462	0.486	8.76
ADF	279.3	242.1	277.4	239.8	278.7	241.9	278.1	244.3	0.042	0.687	0.972	12.4
ADL	57.9	45.9	58.2	46.7	58.5	46.7	58.3	45.9	0.044	0.786	0.341	1.35
Ash	82.1	76.8	83.1	78.3	79.3	79.2	80	76.7	0.753	0.562	0.691	2.14
Starch	205	225.3	203.2	224.9	204.4	223.2	201.1	224.2	0.058	0.642	0.087	5.77
UFL/kg DM	0.89	0.91	0.89	0.91	0.89	0.91	0.89	0.91	0.049	0.452	0.084	0.02
SFA	22.6	23.1	22.8	24	22.6	24.01	22.8	23.8	0.141	0.452	0.132	2.41
MUFA	23.3	20.2	22.9	20.3	23	20.4	23.5	20.1	0.872	0.135	0.245	1.85
PUFA	53.9	55	54.1	54.4	52.98	54.1	53.4	54.6	0.423	0.135	0.136	3.12
ω6	31.8	39.5	31.6	39.1	31.8	39.4	31.9	39	0.048	0.642	0.125	1.21
ω3	21.8	15.4	20.9	15.2	21.7	15.6	21.9	15	0.037	0.521	0.761	2.01

SFA: saturated fatty acids; MUFA: monounsaturated fatty acids; PUFA: polyunsaturated fatty acids. RMSE: root means square error.

**Table 3:** Milk yield (MY kg/head/day) and chemical composition (g/kg) of groups H and L.

	MY		Fat		Protein		Lactose	
	H	L	H	L	H	L	H	L
Mean	11.6	11.7	90.1	85.6	42.5	41.9	46.6	46.6
March	12.1	11.9	80.2	77.1	42.0	42.3	46.7	46.7
April	13.1	13.1	90.6	84.7	42.0	41.0	47.1	46.8
May	11.4	11.6	91.2	88.0	42.5	41.7	46.7	46.5
June	9.9	10.0	100.1	92.6	43.4	42.6	45.9	46.2
	*p* value							
Group	0.658		0.008		0.095		0.752	
Time	0.009		0.006		0.048		0.541	
T × G	0.064		0.089		0.098		0.315	
RMSE	2.694		1.635		0.258		0.212	

RMSE: root means square error.

**Table 4:** Milk fatty acid profile (g/100 g FA) of groups H and L.

	Group		Significance			RMSE
	H	L	G	T	G × T	
C4:0	3.768	3.91	0.937	0.465	0.603	0.737
C6:0	1.755	1.607	0.099	0.049	0.319	0.331
C8:0	1.199	1.009	0.995	0.035	0.743	0.412
C10:0	1.220	1.987	0.045	0.034	0.046	1.263
C11:0	0.092	0.073	0.048	0.321	0.122	0.066
C12:0	1.893	1.610	0.271	0.024	0.104	1.007
C13:0	0.020	0.019	0.598	0.305	0.499	0.063
C14:0	11.30	11.32	0.729	0.241	0.254	1.754
C14:1	1.265	1.514	0.931	0.661	0.705	0.987
C15:0	1.554	1.528	0.303	0.629	0.154	0.853
C15:1 cis-10	0.127	0.137	0.550	0.279	0.579	0.011
C16:0	30.71	30.45	0.173	0.318	0.517	1.698
C16:1	1.396	1.322	0.572	0.839	0.087	0.087
C17:0	1.348	1.266	0.998	0.678	0.070	0.863
C17:1 cis-10	1.208	1.543	0.610	0.026	0.038	0.087
C18:0	10.70	10.80	0.619	0.895	0.340	0.863
C18:1 cis-9	24.07	22.79	0.271	0.035	0.489	1.210
C18:1 trans-9	2.450	2.097	0.805	0.730	0.908	0.865
C18:2 (LA) n6	2.584	2.473	0.284	0.314	0.806	0.653
C18:3n3 (ALA)	0.834	0.540	0.620	0.131	0.730	0.065
C18:3n6 (GLA)	0.098	0.089	0.469	0.614	0.358	0.015
C20:0	0.212	0.229	0.127	0.748	0.954	0.053
C20:1	0.071	0.129	0.813	0.041	0.992	0.021
CLA 9c 11t	0.079	0.054	0.042	0.027	0.110	0.055
CLA 10t 12c	0.022	0.013	0.035	0.041	0.036	0.001
C21:0	0.051	0.035	0.333	0.571	0.180	0.003
C20:3n6	0.060	0.081	0.855	0.791	0.338	0.043
C20:3n3	0.072	0.086	0.439	0.275	0.686	0.026
C23:0	0.025	0.017	0.412	0.882	0.201	0.051
C20:4n6	0.159	0.162	0.084	0.358	0.082	0.021
C22:2n6 cis-13.16	0.030	0.017	0.124	0.218	0.381	0.003
C24:0	0.051	0.032	0.447	0.141	0.574	0.033
C20:5n3	0.099	0.101	0.813	0.029	0.237	0.031
C22:6n3	0.103	0.094	0.305	0.351	0.206	0.042
C22:5n3	0.033	0.028	0.459	0.895	0.315	0.003
SFA	66.11	66.81	0.452	0.021	0.046	1.003
MUFA	29.72	29.54	0.423	0.034	0.021	1.213
PUFA	4.170	3.742	0.042	0.015	0.032	0.213
PUFA N6	2.933	2.820	0.351	0.089	0.352	0.123
PUFA N3	1.142	0.850	0.019	0.026	0.032	0.088
N6/N3	2.570	3.330	0.024	0.037	0.040	0.081
TOTAL CLA	0.101	0.068	0.005	0.023	0.036	0.022

SFA: saturated fatty acids; MUFA: monounsaturated fatty acids; PUFA: polyunsaturated fatty acids. RMSE: root means square error.

**Table 5:** Daily intake (DI, in kg as fed) and cost (EUR/head/day) of ration during the trial of groups H and L.

	DI (kg)		Cost March		Cost April		Cost May		Cost June		SEM	*p* Value
	H	L	H	L	H	L	H	L	H	L		
CS	12.0	20.0	0.96	1.60	0.96	1.60	0.96	1.60	0.96	1.60	-	-
AH	4.5	2.0	0.86	0.38	0.86	0.38	0.86	0.38	0.86	0.38	-	-
PH	4.0	2.0	0.66	0.33	0.66	0.33	0.66	0.33	0.66	0.33	-	-
CM	2.0	2.2	0.60	0.66	0.56	0.62	0.52	0.57	0.51	0.66	-	-
C	3.5	4.7	1.76	2.36	1.67	2.25	1.62	2.18	1.58	2.12	-	-
HF	0.1	0.1	0.17	0.17	0.17	0.17	0.17	0.17	0.17	0.17	-	-
TMR	26.1	32.7	5.01	5.50	4.88	5.34	4.79	5.24	4.73	5.16	0.251	0.01

CS: corn silage; Ah; alfalfa hay; PH, polyphyte hay; CM: corn meal; C: concentrate; HF: hydrogenate fats; TMR: total mixed ration; SEM, standard error of the mean.

**Table 6:** Mozzarella cheese yield (MCY, %) and kg of mozzarella cheese produced by groups H and L.

	H	L	H vs. L/Day	H vs. L/120 Days
MCY (%)	25.1	24.3	0.8	
Mozzarella cheese, kg	58.2	56.8	+1.4	+168

## Data Availability

The data that support the findings of this study are available from the corresponding author upon reasonable request.
